# Insulin resistance and muscle weakness are synergistic risk factors for silent lacunar infarcts: the Bunkyo Health Study

**DOI:** 10.1038/s41598-021-00377-5

**Published:** 2021-10-26

**Authors:** Yuki Someya, Yoshifumi Tamura, Hideyoshi Kaga, Daisuke Sugimoto, Satoshi Kadowaki, Ruriko Suzuki, Shigeki Aoki, Nobutaka Hattori, Yumiko Motoi, Kazunori Shimada, Hiroyuki Daida, Muneaki Ishijima, Kazuo Kaneko, Shuko Nojiri, Ryuzo Kawamori, Hirotaka Watada

**Affiliations:** 1 Sportology Center, Juntendo University Graduate School of Medicine, Tokyo, Japan; 2 Juntendo University Graduate School of Health and Sports Science, Chiba, Japan; 3 Department of Metabolism & Endocrinology, Juntendo University Graduate School of Medicine, 2-1-1 Hongo, Bunkyo-ku, Tokyo, 113-8421 Japan; 4 Department of Radiology, Juntendo University Graduate School of Medicine, Tokyo, Japan; 5 Department of Neurology, Juntendo University Graduate School of Medicine, Tokyo, Japan; 6 Department of Diagnosis Prevention and Treatment of Dementia, Juntendo University Graduate School of Medicine, Tokyo, Japan; 7 Department of Cardiovascular Medicine, Juntendo University Graduate School of Medicine, Tokyo, Japan; 8 Department of Medicine for Orthopaedics and Motor Organ, Juntendo University Graduate School of Medicine, Tokyo, Japan; 9 Medical Technology Innovation Center, Juntendo University, Tokyo, Japan

**Keywords:** Risk factors, Diabetes, Pre-diabetes, Stroke

## Abstract

Insulin resistance and muscle weakness are risk factors for silent lacunar infarcts (SLI), but it is unclear whether they are still independent risk factors when adjusted for each other. In addition, the effect of their combination on SLI is completely unknown. We evaluated SLI, insulin sensitivity, and knee extensor muscle strength by magnetic resonance imaging, PREDIM, and dynamometer, respectively, in 1531 elderly people aged 65–84 years living in an urban area of Tokyo. Among the study subjects, 251 (16.4%) had SLI. Impaired insulin sensitivity (High; 1.00 [reference], Medium; 1.53 [95% confidence interval (CI) 0.94–2.48], Low; 1.86 [1.02–3.39], p for trend 0.047) and reduced muscle strength (High; 1.00 [reference], Medium; 1.40 [0.98–2.02], Low; 1.49 [1.04–2.15], p for trend 0.037) were independently associated with increased risk for SLI in the fully adjusted model. In terms of combined, subjects classified as having the lowest insulin sensitivity and lowest strength were 4.33 times (95% CI 1.64–11.45) more likely to have a SLI than those classified as having the highest insulin sensitivity and highest strength. Impaired insulin sensitivity and reduced muscle strength were independently associated with higher risk of SLI in elderly subjects, and their combination synergistically increased this risk.

## Introduction

Lacunar infarcts are often incidentally found on brain magnetic resonance imaging (MRI) scans in asymptomatic elderly people, and in such cases they are defined as silent lacunar infarcts. Some epidemiological studies revealed that over a quarter of elderly Japanese people have silent brain infarcts^1–4^, and it has been shown that they are a risk factor for fatal and non-fatal stroke^5,6^, dementia^7^ and frailty^8^. In particular, subjects with silent lacunar infarcts in a Japanese cohort had a 10.48-fold increased risk of incident stroke^6^. Therefore, prevention of silent lacunar infarcts might be an important strategy to prevent future stroke, dementia, and frailty in elderly subjects, especially in subjects with diabetes because of their increased risk of stroke, dementia, and frailty^9^. However, the overall etiology of silent brain infarcts remains unclear, though aging and hypertension are recognized as reliable risk factors^1,10^.

Several studies have shown that metabolic syndrome is strongly associated with silent brain infarcts^1,11^, increasing their risk by 2.4-fold in one report^11^. Because insulin resistance is the fundamental condition underlying the pathogenesis of metabolic syndrome^12,13^, it is also a risk factor for silent lacunar infarcts. In fact, Lee et al. demonstrated in a cross-sectional study that subjects with insulin resistance, defined as over 2.56 times the cutoff of the homeostasis model assessment of insulin resistance (HOMA-IR), had a 1.69-fold higher risk of silent lacunar infarcts than subjects without insulin resistance^14^. No other research has investigated the direct relationship between insulin resistance and silent lacunar infarcts.

Several studies, however, have demonstrated that reduced muscle strength is a risk factor for silent lacunar infarcts. Indeed, it was reported that low muscle strength at age 18 years was a risk factor for future ischemic stroke^15^. Lower handgrip strength in middle age also increased the risk of cerebral infarction^16^. Very recently, we revealed that reduced muscle strength was associated with a higher risk for silent lacunar infarcts^17^. Given that skeletal muscle is a major contributor to the whole-body insulin sensitivity as measured by the hyperinsulinemic euglycemic clamp^13^ or surrogate methods such as PREDIM^18^, impaired muscle functions such as insulin resistance and reduced muscle strength seem to be risk factors for silent lacunar infarcts. However, it is unclear whether they act independently of one another, since insulin sensitivity is weakly but positively associated with muscle strength^19,20^. In addition, even though muscle strength and insulin sensitivity are independent risk factors, it remains unclear how their combinations predict the incidence of silent lacunar infarcts.

Given the above background, the present study investigated whether reduced muscle strength and insulin sensitivity are independent risk factors for silent lacunar infarcts, using the data of 1531 elderly subjects aged 65–84 years who participated in the Bunkyo Health Study. In addition, we evaluated the combined effect of insulin sensitivity and muscle strength on silent lacunar infarcts.

## Results

### Characteristics according to insulin sensitivity groups

Of the 1531 participants in this cohort, 251 (16.4%) had silent lacunar infarcts. The mean age of participants was 73.0 ± 5.4 years. The mean insulin sensitivity value (PREDIM) was 6.09 ± 2.18 in men and 6.90 ± 2.43 in women. The cut-off value for low insulin sensitivity (under − 1SD) was similar to that in Japanese diabetic patients^21,22^. Characteristics of the subjects in the three groups divided according to insulin sensitivity are shown in Table 1. The prevalence of silent lacunar infarcts was inversely associated with insulin sensitivity. Reduced insulin sensitivity was associated with higher age, higher BMI, lower physical activity, and lower muscle strength. Similarly, we observed associations between reduced insulin sensitivity and higher prevalences of cognitive impairment, hypertension, diabetes, dyslipidemia, and cardiovascular disease. Most of trends in each parameter such as prevalence of silent lacunar infarcts were similar between the classifications, while several parameters such as HbA1c, liver function test and total adiponectin showed different trends (Supplementary Table S1).

**Table 1:** Characteristics according to insulin sensitivity categories.

*N* (M/F)	All	High (+ 1SD)	Medium	Low (− 1SD)	p for trend*
	1531 (627/904)	236 (96/140)	1057 (431/626)	238 (100/138)	
PREDIM	6.6 ± 2.4	10.5 ± 1.7	6.4 ± 1.3	3.5 ± 0.6	
Silent lacunar infarcts	251 (16.4%)	23 (9.7%)	174 (16.5%)	54 (22.7%)	< 0.001
Age, years	73.0 ± 5.4	71.9 ± 5.3	73.0 ± 5.3	74.1 ± 5.3	< 0.001
Height, cm	158.0 ± 8.8	158.1 ± 8.3	158.1 ± 8.7	157.3 ± 9.4	0.354
Body weight, kg	56.9 ± 10.2	49.2 ± 8.2	57.1 ± 9.3	64.1 ± 10.7	< 0.001
Body mass index, kg/m^2^	22.7 ± 3.0	19.6 ± 2.1	22.7 ± 2.4	25.8 ± 3.1	< 0.001
Skeletal muscle index, kg/m^2^	6.4 ± 1.0	6.1 ± 0.9	6.4 ± 0.9	6.7 ± 1.0	< 0.001
Percent body fat, %	28.3 ± 7.3	21.1 ± 5.6	28.4 ± 6.2	34.6 ± 6.7	< 0.001
Waist circumference, cm	86.5 ± 9.2	78.2 ± 7.2	86.5 ± 7.8	94.7 ± 9.5	< 0.001
Hypertension	991 (64.7%)	109 (46.2%)	680 (64.3%)	202 (84.9%)	< 0.001
Diabetes	186 (12.1%)	2 (0.8%)	79 (7.5%)	105 (44.1%)	< 0.001
Hyperlipidemia	957 (62.5%)	100 (42.4%)	660 (62.4%)	197 (82.8%)	< 0.001
Cardiovascular disease	67 (4.4%)	1 (0.4%)	48 (4.5%)	18 (7.6%)	< 0.001
Cognitive impairment	258 (16.9%)	37 (15.7%)	164 (15.5%)	57 (23.9%)	0.016
**Smoking**					
Past smoking	500 (32.7%)	77 (32.6%)	338 (32.0%)	85 (35.7%)	0.472
Current smoking	114 (7.4%)	25 (10.6%)	80 (7.6%)	9 (3.8%)	0.005
Muscle strength, Nm	76.5 ± 27.3	71.6 ± 22.6	77.5 ± 27.4	76.6 ± 30.3	0.203
Muscle strength, Nm/kg	133.7 ± 37.4	144.6 ± 34.1	134.8 ± 37.2	118.1 ± 36.6	< 0.001
Physical activity, METs/h/week	30.5 (16.5–54.2)	37.2 (20.6–67.7)	29.7 (16.5–51.6)	26.5 (13.4–49.7)	< 0.001
Sedentary time, h	6.0 ± 3.6	5.8 ± 3.5	6.0 ± 3.6	6.4 ± 3.6	0.072
Dietary intake, kcal	1963.6 ± 594.1	1979.2 ± 593.7	1966.4 ± 590.0	1936.6 ± 614.0	0.387
Protein intake, g	83.1 ± 30.6	81.4 ± 29.9	83.2 ± 30.2	84.3 ± 33.1	0.502
Fat intake, g	61.6 ± 22.0	59.5 ± 21.0	61.8 ± 21.9	63.0 ± 23.0	0.101
Carbohydrate intake, g	242.5 ± 82.5	250.5 ± 82.5	242.3 ± 82.0	235.6 ± 84.1	0.013
Salt, g	12.5 ± 4.1	12.2 ± 3.9	12.5 ± 4.0	12.8 ± 4.4	0.204
Alcohol, g	1.0 (0.0–17.1)	2.5 (0.0–18.0)	1.1 (0.0–17.7)	0.2 (0.0–9.7)	0.002
Systolic blood pressure, mmHg	136.4 ± 17.1	131.3 ± 18.6	136.4 ± 17.0	141.5 ± 14.2	< 0.001
Diastolic blood pressure, mmHg	84.3 ± 9.7	81.3 ± 10.0	84.5 ± 9.7	86.1 ± 8.9	< 0.001
Fasting plasma insulin, μU/mL	4.1 (2.8–5.9)	2.1 (1.6–2.7)	4.1 (3.0–5.4)	7.8 (5.9–11.2)	< 0.001
Fasting plasma glucose, mg/dL	96.0 (90.0–104.0)	90.0 (86.0–94.0)	96.0 (91.0–102.0)	112.5 (102.0–135.3)	< 0.001
HbA1c, %	5.8 ± 0.6	5.5 ± 0.3	5.8 ± 0.4	6.4 ± 0.8	< 0.001
Total cholesterol, mg/dL	207.3 ± 36.0	212.2 ± 32.7	208.1 ± 36.3	199.0 ± 36.7	< 0.001
LDL cholesterol, mg/dL	122.1 ± 30.6	123.1 ± 28.9	122.8 ± 30.8	118.1 ± 31.2	0.057
HDL cholesterol, mg/dL	64.7 ± 16.6	72.4 ± 16.2	64.8 ± 16.2	56.6 ± 14.9	< 0.001
Triglycerides, mg/dL	85.0 (64.0–117.0)	68.0 (52.3–88.8)	84.0 (64.5–115.0)	113.0 (85.8–157.3)	< 0.001
Aspartate aminotransferase, IU/L	22.0 (19.0–25.0)	23.0 (20.0–26.0)	21.0 (19.0–25.0	23.0 (20.0–28.0)	0.705
Alanine aminotransferase, IU/L	17.0 (13.0–21.0)	15.0 (12.0–19.0)	16.0 (13.0–21.0)	20.0 (17.0–29.0)	< 0.001
γ-Glutamyl transferase, IU/L	21.0 (16.0–33.0)	18.0 (13.0–26.0)	21.0 (16.0–32.0)	30.0 (20.0–46.0)	< 0.001
Serum albumin, g/dL	43.0 ± 3.7	42.6 ± 4.3	43.0 ± 3.6	43.4 ± 3.8	0.033
Creatinine, mg/dL	0.73 (0.62–0.86)	0.73 (0.61–0.85)	0.73 (0.62–0.86)	0.73 (0.63–0.85)	0.515
Total adiponectin, μg/mL	10.9 (7.7–15.5)	15.5 (11.3–20.9)	10.8 (7.8–15.1)	8.2 (6.1–10.8)	< 0.001
25-Hydroxyvitamin D, ng/mL	19.1 ± 5.1	19.3 ± 5.7	19.2 ± 5.1	18.6 ± 4.7	0.197

Data are mean ± SD, mean (IQR), or number (%).

*p for trend was analyzed by the Jonckheere Terpstra test (continuous variables) and Cochran-Armitage trend test (categorical variables).

### Insulin sensitivity, muscle strength, and silent lacunar infarcts

**Figure 1:**
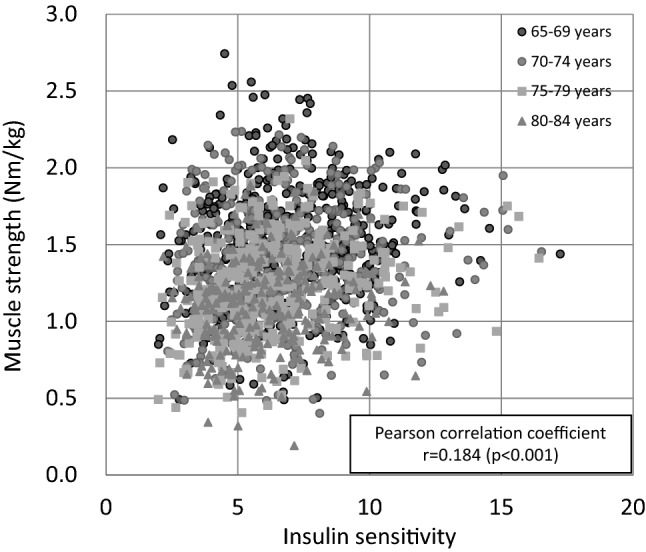
Correlation between insulin sensitivity and muscle strength.

Insulin sensitivity was weakly but significantly correlated with muscle strength (Fig. 1, r = 0.184, p < 0.001). Table 2 shows the OR for silent lacunar infarcts in each group. The age- and sex-adjusted model revealed that both reduced insulin sensitivity and reduced muscle strength were significantly associated with a higher OR for silent lacunar infarcts (insulin sensitivity: High, 1.00 [reference]; Medium, 1.67 [95% CI 1.05–2.67]; Low: 2.24 [95% CI 1.31–3.84], p for trend 0.003; muscle strength: High, 1.00 [reference]; Medium, 1.48 [95% CI 1.03–2.11]; Low: 1.65 [95% CI 1.16–2.35], p for trend 0.006). Even after adjustment for risk factors such as muscle strength (model 3), the lowest insulin sensitivity group (under -1SD) was associated with a higher OR for silent lacunar infarcts (High: 1.00 [reference]; Medium, 1.53 [95% CI 0.94–2.48]; Low, 1.86 [95% CI 1.02–3.39]). Furthermore, the linear trend across the three groups was significant (p for trend: 0.047). Additionally, the lowest muscle strength group was associated with a higher OR for silent lacunar infarcts even with adjustment for insulin sensitivity and other parameters in model 3 (High, 1.00 [reference]; Medium, 1.40 [95% CI 0.98–2.02]; Low: 1.49 [95% CI 1.04–2.15], p for trend 0.037). These data demonstrate that impaired insulin sensitivity and reduced muscle strength are each independently associated with higher risk for silent lacunar infarcts.

**Table 2:** Associations between insulin sensitivity and silent lacunar infarcts.

	Odds (95% CI)		
	Model 1	Model 2	Model 3
Insulin sensitivity			
High (≥ 1SD)	1.00	1.00	1.00
Medium	1.67 (1.05–2.67)	1.64 (1.02–2.63)	1.53 (0.94–2.48)
Low (≤ -1SD)	2.24 (1.31–3.84)	2.06 (1.19–3.58)	1.86 (1.02–3.39)
p for trend	0.003	0.011	0.047
Muscle strength			
High	1.00	1.00	1.00
Medium	1.48 (1.03–2.11)	1.39 (0.97–2.00)	1.40 (0.98–2.02)
Low	1.65 (1.16–2.35)	1.53 (1.06–2.19)	1.49 (1.04–2.15)
p for trend	0.006	0.025	0.037
Age (per 1 year)	1.11 (1.09–1.14)	1.10 (1.07–1.13)	1.09 (1.06–1.13)
Sex			
Male	1.00	1.00	1.00
Female	0.88 (0.66–1.17)	0.79 (0.54–1.16)	0.89 (0.61–1.29)
Smoking			
Never		1.00	1.00
Past		1.06 (0.74–1.52)	1.09 (0.75–1.55)
Current		0.67 (0.34–1.34)	0.69 (0.35–1.40)
Physical activity (per METs/h/week)		1.00 (1.00–1.00)	1.00 (1.00–1.00)
Hypertension (yes)			1.82 (1.29–2.57)
Diabetes (yes)			0.92 (0.59–1.44)
Hyperlipidemia (yes)			0.87 (0.64–1.18)
Cardiovascular disease (yes)			1.87 (1.06–3.30)

Model 1 was adjusted for age and sex.

Model 2 was adjusted for muscle strength or insulin sensitivity, smoking, physical activity, and incorporated model 1.

Model 3 was adjusted for hypertension, diabetes, dyslipidemia, cardiovascular disease, and incorporated model 2.

### Combination of muscle strength and muscle insulin sensitivity

**Figure 2:**
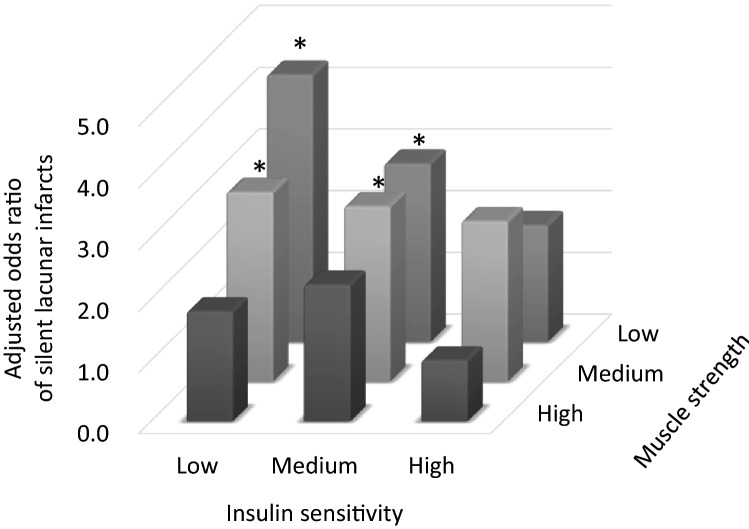
Interaction between insulin sensitivity and muscle strength in relation to silent lacunar infracts. Odds ratios were adjusted for age, sex, smoking, daily physical activity, hypertension, diabetes, dyslipidemia, and cardiovascular disease. *p < 0.05 compared with the category with the highest insulin sensitivity and muscle strength.

Next, to investigate the combined effect of reduced muscle strength and insulin sensitivity on the risk for silent lacunar infarcts, the participants were categorized into nine groups according to combinations of insulin sensitivity and muscle strength, and the OR for silent lacunar infarcts in each group is shown in Fig. 2. After adjusting for age, hypertension, and other potential risk factors, OR for silent lacunar infarcts was significantly higher in 4 groups with either insulin sensitivity or muscle strength was not “High” compared with the group with High-insulin sensitivity and High-muscle strength. Especially, subjects with the Low-insulin sensitivity and Low-muscle strength were 4.33 times (95% CI 1.64–11.45) more likely to have silent lacunar infarcts than those with the High-insulin sensitivity and High-muscle strength. Furthermore, Low-insulin sensitivity combined with Low-muscle strength had a sensitivity of 68.5%, specificity of 63.0%, positive predictive values of 26.7%, negative predictive values of 91.1% (area under the receiver operating characteristic curve of 0.71 [95% CI 0.67–0.74]) for silent lacunar infarcts. These results suggest that insulin resistance and reduced muscle strength synergistically increase the risk for silent lacunar infarcts.

### Insulin sensitivity, muscle strength, and silent lacunar infarcts in each gender

We preliminary performed logistic regression analysis in each gender. As shown in Supplementary Tables S2 and S3, the association of muscle strength and silent lacunar infarcts was significant in male, but not in female, while the association between insulin sensitivity and silent lacunar infarcts in each gender showed similar trend, but statistically non-significant probably due to small number of subjects. In terms of combination effect of muscle strength and muscle insulin sensitivity, subjects with the Low-insulin sensitivity and Low-muscle strength were higher OR for silent lacunar infarcts compared with those with the High-insulin sensitivity and High-muscle strength in each gender; however, the OR in male was higher than that in female (male, 11.40 [95% CI 1.30–100.24]; female, 3.46 [1.12–10.67]) (Supplementary Fig. S1).

## Discussion

In this study, we investigated whether reduced insulin sensitivity and muscle strength were independent risk factors for silent lacunar infarcts in 1531 elderly subjects living in an urban area in Tokyo, Japan. We also evaluated the combined effect of insulin sensitivity and muscle strength on silent lacunar infarcts. In this cohort, 251 (16.4%) subjects had silent lacunar infarcts. After full adjustment for potential risk factors, we found that impaired insulin sensitivity and reduced muscle strength were independently associated with an increased risk for silent lacunar infarcts. Furthermore, we found that subjects with the lowest insulin sensitivity and muscle strength had a 4.33-fold (95% CI 1.64–11.45) higher risk for silent lacunar infarcts than those in the groups with the highest values for both parameters.

This study showed that lower insulin sensitivity evaluated by PREDIM was independently associated with higher risk of silent lacunar infarcts after full adjustment for potential risk factors. Most previous epidemiological studies used HOMA-IR as an index of insulin sensitivity; however, the accuracy of the index remains questionable. For example, Kang et al. suggested that the correlation between HOMA-IR and the glucose disposal rate measured by hyperinsulinemic euglycemic clamp is lower in subjects with lower BMI, lower beta cell function, and higher fasting glucose levels, such as lean individuals with type 2 diabetes mellitus with insulin secretory defects^23^. Thus, several studies excluded patients with diabetes mellitus due to inaccurate estimation of insulin resistance by HOMA-IR^24–26^. Of note, one such study demonstrated that insulin resistance evaluated by HOMA-IR was independently associated with silent lacunar infarcts^14^. Based on this background, we used PREDIM as the index of insulin sensitivity since its accuracy has been validated in subjects with broad ranges of BMI, age, and glycemic control, including diabetic patients, and we successfully demonstrated the association between insulin resistance and silent lacunar infarcts and included individuals with diabetes mellitus.

This study also showed that impaired insulin sensitivity and reduced muscle strength were independently associated with increased risk for silent lacunar infarcts, and the two factors demonstrated a significant combined effect. PREDIM is correlated with the glucose infusion rate during hyperinsulinemic euglycemic clamp^18^, mainly reflecting insulin sensitivity in muscle^13^. Thus, the present study suggested that two muscle characteristics, strength and insulin sensitivity, are independently and synergistically associated with silent lacunar infarcts. From a biological viewpoint, muscle strength and muscle insulin sensitivity are regulated differently. It has been reported that reduced muscle strength is caused by several defects, such as neurological dysfunction^27^, impaired Ca^2+^ handling in myocytes^28^ and reduced muscle mass^29^. On the other hand, muscle insulin resistance has been reported to be induced by different defects, such as impaired insulin signal transduction due to low-grade inflammation and intramyocellular lipid accumulation^30^. In fact, similar to previous studies^19,20^, the present study revealed only a weak overall association between muscle strength and insulin sensitivity, and this association varied widely between individuals (Fig. 1), suggesting a different etiology of reduced muscle strength and insulin resistance.

The underlying mechanisms linking impaired insulin sensitivity, reduced muscle strength, and silent lacunar infarcts are not yet fully understood. Atherosclerosis has been recognized as a cause of silent lacunar infarcts^31^, and it has been suggested that insulin resistance induces atherosclerosis^32^. Thus, lower insulin sensitivity might cause silent lacunar infarcts via atherosclerosis. In terms of muscle strength, previous prospective studies showed that reduced muscle strength at 18 years of age was associated with an increased risk of ischemic stroke later in life^15,33^. Thus, by an undefined mechanism, lower muscle strength is associated with silent lacunar infarcts. Alternatively, it is also possible that silent lacunar infarcts cause lower muscle strength. In fact, patients with silent lacunar infarcts were shown to have diminished physical functions, for instance slow gait speed, loss of balance, and low physical activity^34–36^. Presence of silent lacunar infarctions was also associated with minor neuropsychological alterations, such as executive functions (semantic fluency) and short delayed verbal memory^37^.

The current study has several limitations. This cohort included only participants living in an urban part of Japan, and relatively healthy participants may have been included. However, the prevalence rate of silent lacunar infarcts (16.4%) in the present study was similar to that in a previous study in Japanese^1^, and thus our data should be reflective of elderly individuals living in an urban area of Japan. In addition, this study was cross-sectional in nature, and thus causal relationships have not yet been determined. Further prospective and interventional studies are required to clarify the roles of muscle insulin sensitivity and strength on the incidence of silent lacunar infarcts. Finally, elderly subjects with reduced insulin sensitivity could have other comorbidities which confounded the association between insulin sensitivity and silent lacunar infarcts.

In conclusions, impaired insulin sensitivity and reduced muscle strength were independently associated with a higher risk for silent lacunar infarcts, and the combination of both synergistically elevated this risk in Japanese elderly individuals.

## Methods

### Study design and participants

The Bunkyo Health Study is a prospective cohort study designed to clarify how muscle mass, muscle strength, and insulin sensitivity are associated with multiple diseases that necessitate long-term care^38^. In this study, we recruited elderly subjects aged 65–84 years living in Bunkyo-ku, an urban area in Tokyo, Japan. All subjects participated in examinations over two visits to the Sportology Center from October 15, 2015, to October 1, 2018. Briefly, we evaluated cognitive function by questionnaires, physical fitness by dynamometer and physical performance tests, brain lesions by MRI, body composition and bone mineral density by dual-energy X-ray absorptiometry (DXA), arteriosclerosis by the cardio-ankle vascular index (CAVI), and abdominal fat distribution by MRI. We also carried out a 75-g oral glucose tolerance test (OGTT). This study protocol was approved by the ethics committee of Juntendo University in November 2015 (Nos. 2015078, 2016138, 2016131, 2017121, and 2019085). This study is carried out in accordance with the principles outlined in the Declaration of Helsinki. All participants gave written informed consent at the orientation meeting. Participants were told that they had the right to withdraw from the trial at any time.

This study enrolled 1612 subjects who had undergone OGTT, muscle strength testing, and brain MRI at the beginning of the Bunkyo Health Study. Missing data were relatively infrequent for OGTT (0.3%), muscle strength (0.3%), and MRI (0.4%). Among the 1612 subjects, 81 with a previous stroke event were excluded; these subjects either had a previous confirmed clinical stroke (n = 52) or newly found non-lacunar brain infarcts by MRI in the Bunkyo Health Study (n = 29). Therefore, 1531 subjects were analyzed.

### Evaluation of insulin sensitivity

Insulin sensitivity was estimated by PREDIM, a recently established index for insulin sensitivity^18^. PREDIM was calculated from the Oral Glucose Insulin Sensitivity (OGIS) index^39^ and other parameters, including body mass index (BMI), 2-h glucose during OGTT, and fasting insulin, to achieve good correlation with insulin sensitivity (*M* value) measured by hyperinsulinemic euglycemic clamp.

The OGIS is calculated using the following equation^39^:

$${\text{OGIS}} = {1}/{2} \times \left( {B + {\text{square }}\left( {B^{{2}} + {4} \times {\text{p5}} \times {\text{p6}} \times \left( {{\text{Glu9}}0 - {\text{Glu}}_{{{\text{CLAMP}}}} } \right) \, \times {\text{Cl}}_{{{\text{OGTT}}}} } \right)} \right),$$

$$B = \left( {{\text{p5}} \times \left( {{\text{Glu9}}0 - {\text{Glu}}_{{{\text{CLAMP}}}} } \right) + {1}} \right) \, \times {\text{Cl}}_{{{\text{OGTT}}}} ,$$

$${\text{Cl}}_{{{\text{OGTT}}}} = {\text{p4}} \times \left( {\left( {{\text{p1}} \times {\text{D}}_{{\text{O}}} - {\text{V}} \times \left( {{\text{Glu12}}0 - {\text{Glu9}}0} \right)/{3}0} \right)/{\text{Glu9}}0 + {\text{p3}}/{\text{Glu}}0} \right)/\left( {{\text{Ins9}}0 - {\text{Ins}}0 + {\text{p2}}} \right).$$

*Glu0, Glu90, Glu120: glucose concentration values (mmol/L), Ins0, Ins90: insulin concentration values (pmol/L), D_O_: glucose dose of the OGTT (mmol/m^2^, i.e. normalized for body surface area), V: glucose distribution volume = 10^4^ ml/kg, Glu_CLAMP_: clamp glucose concentration = 5 mmol/L, p1 = 6.50, p2 = 1951, p3 = 4514, p4 = 792, p5 = 11.8 × 10^–3^, p6 = 173.

PREDIM is calculated using the following equation^18^:

$${\text{log}}_{{\text{e}}} \left( {{\text{PREDIM}}} \right) = {\text{A}} + {\text{B}} \times {\text{log}}_{{\text{e}}} \left( {{\text{OGIS}}} \right) + {\text{C}} \times {\text{log}}_{{\text{e}}} \left( {{\text{BMI}}} \right) + {\text{D}} \times {\text{log}}_{{\text{e}}} \left( {{\text{Glu12}}0} \right) + {\text{E}} \times {\text{log}}_{{\text{e}}} \left( {{\text{Ins}}0} \right).$$

*Glu120: glucose concentration values (mmol/L), Ins0: insulin concentration values (pmol/L), A = 2.8846219, B = 0.5208520, C = − 0.8223363, D = − 0.4191242, E = − 0.2427896.

The equivalence test in subgroups showed that the clamp-derived *M* value and PREDIM value were similar for comparison of subgroups based on glucose tolerance (normal glucose tolerance, impaired fasting glucose, impaired glucose tolerance, type 2 diabetes) and BMI (lean, overweight, obesity)^18^.

### Muscle strength measurement

We evaluated the isokinetic muscle strength of the knee extensors using a dynamometer (BIODEX system 3 or 4: Biodex Medical Systems, Upton, NY, USA)^17^. Participants were stabilized in the examination chair with shoulder and abdominal straps. The isokinetic peak torques of the knee extensors were measured at an angular velocity of 60 degrees per second. During the test, participants were encouraged to exert maximal muscle force. The isokinetic peak torques of the knee extensors were adjusted by body weight according to the following formula: isokinetic peak torques (Nm)/body weight (kg)^17^.

### Evaluation of silent lacunar infarcts

The whole brain of each subject was scanned with a 0.3-T clinical MR scanner (AIRIS Vento, Hitachi, Tokyo, Japan). Sequences included axial three-dimensional (3D) time-of-flight magnetic resonance angiography (repetition time (TR), 35 ms; echo time (TE), 7.1 ms; and slice thickness, 1.2 mm), T2*-weighted gradient echo (T2*-WI) imaging (TR, 1000 ms; TE, 45 ms; flip angle, 20°; and slice thickness, 5 mm) and fluid-attenuated inversion recovery (FLAIR) imaging (TR, 11,000 ms; TE, 100 ms; inversion time (TI), 2000 ms; and slice thickness, 5 mm). The evaluation of lacunar infarcts was conducted by an experienced neuroradiologist based on axial T2*-WI and FLAIR images. The radiologist was blinded to all clinical data. Lacunar infarcts were defined as infarcts of 3–15 mm in diameter located in the deep cerebral white matter, basal ganglia, or pons, and that were presumed to result from the occlusion of a single, small, perforating artery supplying the subcortical area of the brain^31,40^.

### Other measurements

BMI was calculated as weight in kilograms divided by the square of height in meters (kg/m^2^). Physical activity was evaluated using the International Physical Activity Questionnaire (IPAQ), which assesses different types of physical activity, such as walking and moderate- and high-intensity activities^41,42^. Cognitive function was primarily evaluated using the Montreal Cognitive Assessment (MoCA)^43,44^. The MoCA contain 9 items and possible scores range from 0 to 30 points. In this study, we used MoCA score ≤ 22 as the cut-off for cognitive impairment^45,46^. Hypertension was defined as systolic blood pressure (SBP) ≥ 140 mmHg and/or diastolic blood pressure (DBP) ≥ 90 mmHg or current treatment with antihypertensive medications. Diabetes was defined as a combination of fasting plasma glucose ≥ 126 mg/dL and/or a 2-h glucose level ≥ 200 mg/dL after the 75-g OGTT, and hemoglobin A1c ≥ 6.5%, or currently treatment with medication for diabetes mellitus. Dyslipidemia was defined as low-density lipoprotein cholesterol (LDL-C) ≥ 140 mg/dL and/or high-density lipoprotein cholesterol (HDL-C) < 40 mg/dL and/or triglycerides (TG) ≥ 150 mg/dL, or current treatment with lipid-lowering agents. Cardiovascular disease was defined by a medical history of ischemic heart disease or heart failure.

### Statistical analysis

Male and female participants were separately divided into three groups (High, Medium, and Low) based on the standard deviation (SD) of sex-specific values of PREDIM and divided three groups (High, Medium, and Low) based on the tertiles of sex and age (65–69, 70–74, 75–79, and 80–84 years)-specific value of muscle strength (Table 3). Then, participants of both sexes were categorized into nine groups according to the combination of these values. Data are presented as mean ± SD or number (%). Characteristics of the three insulin sensitivity groups were analyzed for trend by the Jonckheere–Terpstra test (continuous variables) and Cochran–Armitage trend test (categorical variables). All statistical tests were two-sided with a 5% significant level. Logistic regression analysis was used to estimate odds ratios (ORs) and 95% confidence intervals (CIs) for the associations between the prevalence of silent lacunar infarcts and insulin sensitivity, muscle strength, and their combination, with adjustment for age (continuous variable), sex (male or female), and other potential risk factors. In this study, three models were applied for regression analysis: model 1 was adjusted for age and sex; model 2 was adjusted for muscle strength (continuous variable) or insulin sensitivity (continuous variable), physical activity level (continuous variable), and cigarette smoking (never, past and current smoker), and incorporated model 1; and model 3 was adjusted for hypertension (yes or no), diabetes (yes or no), dyslipidemia (yes or no), and cardiovascular disease (yes or no), and incorporated model 2.

**Table 3:** Cut-off values for insulin sensitivity and muscle strength.

	Male			Female		
	High	Medium	Low	High	Medium	Low
**A. Insulin sensitivity**						
65–84 years	≥ 8.29	8.29–3.90	≤ 3.90	≥ 9.34	9.34–4.47	≤ 4.47
**B. Muscle strength (Nm/kg)**						
65–69 years	≥ 1.83	1.83–1.53	≤ 1.53	≥ 1.50	1.50–1.23	≤ 1.23
70–74 years	≥ 1.70	1.70–1.40	≤ 1.40	≥ 1.39	1.39–1.13	≤ 1.13
75–79 years	≥ 1.50	1.50–1.25	≤ 1.25	≥ 1.27	1.27–1.00	≤ 1.00
80–84 years	≥ 1.36	1.36–1.10	≤ 1.10	≥ 1.17	1.19–0.90	≤ 0.90

A. Categorized for three groups based on the standard deviation (SD) of sex (male/female)-specific value of PREDIM.

B. Categorized for three groups based on the tertile of age (65–69, 70–74, 75–79, 80–84) and sex (male/female)-specific value of knee extensor muscle strength.

## Supplementary Information

Supplementary Legends.

Supplementary Figure S1.

Supplementary Table S1.

Supplementary Table S2.

Supplementary Table S3.
