# Supplementary material for: Insulin resistance and muscle weakness are synergistic risk factors for silent lacunar infarcts: the Bunkyo Health Study
Source: Sci Rep. 2021 Oct 26;11:21093. doi: 10.1038/s41598-021-00377-5 (PMC8548532; doi:10.1038/s41598-021-00377-5)
Supplement: Supplementary file 1 — Supplementary Legends. [file 41598_2021_377_MOESM1_ESM.docx]

**Supplementary Fig. 1. Interaction between insulin sensitivity and muscle strength in relation to silent lacunar infracts in each gender**

Odds ratios were adjusted for age, smoking, daily physical activity, hypertension, diabetes, dyslipidemia, and cardiovascular disease.

*p<0.05 compared with the category with the highest insulin sensitivity and muscle strength
