# Supplementary figures and images for: Insulin resistance and muscle weakness are synergistic risk factors for silent lacunar infarcts: the Bunkyo Health Study
Source: Sci Rep. 2021 Oct 26;11:21093. doi: 10.1038/s41598-021-00377-5 (PMC8548532; doi:10.1038/s41598-021-00377-5)

## Slide 1
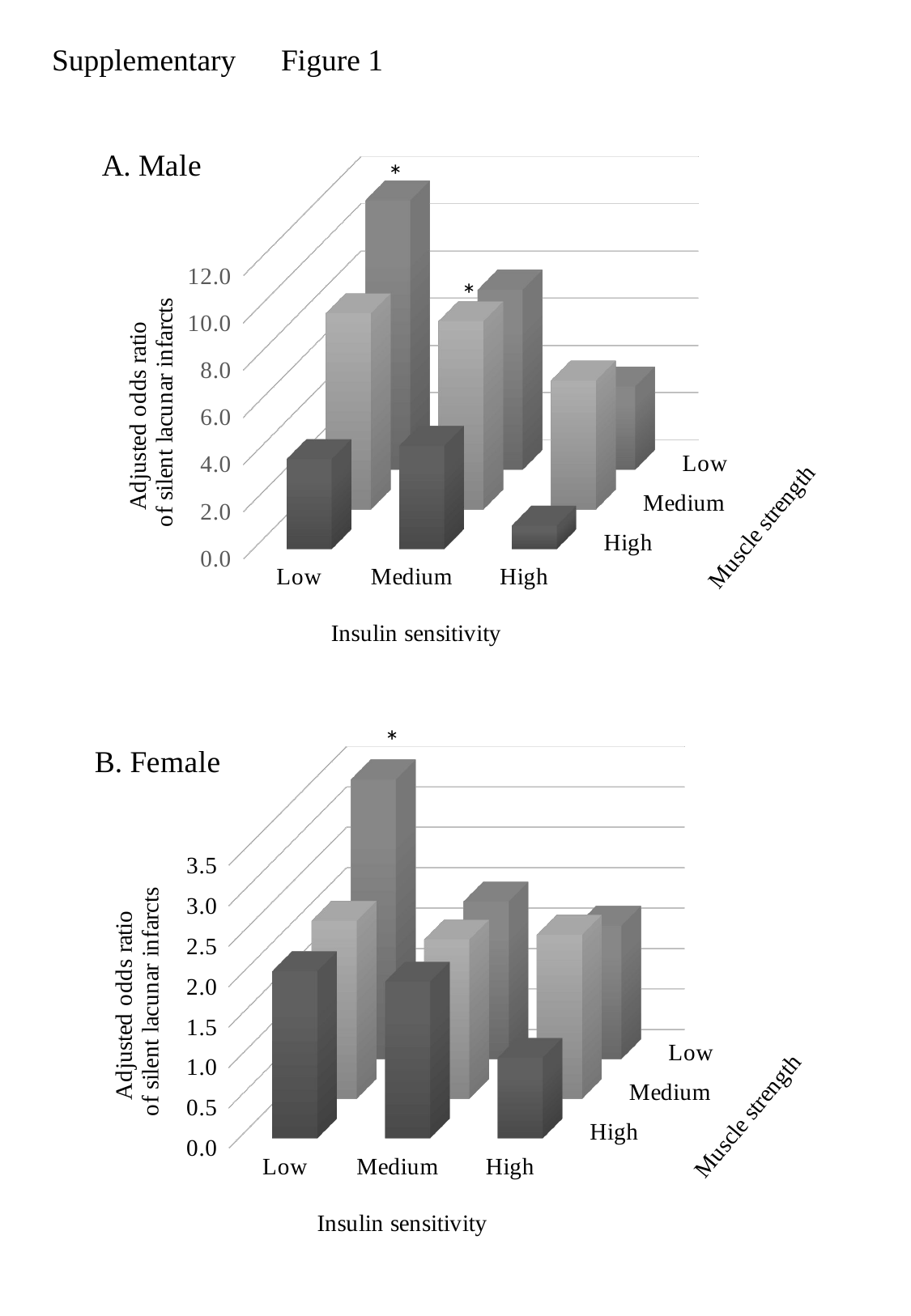

Supplementary　Figure 1
[unsupported chart]
*
*
[unsupported chart]

Supplement: Supplementary file 2 — Supplementary Figure S1. [file 41598_2021_377_MOESM2_ESM.pptx]
