# Supplementary material for: Insulin resistance and muscle weakness are synergistic risk factors for silent lacunar infarcts: the Bunkyo Health Study
Source: Sci Rep. 2021 Oct 26;11:21093. doi: 10.1038/s41598-021-00377-5 (PMC8548532; doi:10.1038/s41598-021-00377-5)
Supplement: Supplementary file 3 — Supplementary Table S1. [file 41598_2021_377_MOESM3_ESM.docx]

**Supplementary Table 1. Characteristics according to muscle strength categories**

|  | All | High | Medium | Low | p for trend* |
| --- | --- | --- | --- | --- | --- |
| *N*  *(M/F)* | 1531  (627/904) | 511  (211/300) | 512  (208/304) | 508  (208/300) |  |
| PREDIM | 6.6±2.4 | 7.3±2.4 | 6.4±2.3 | 6.0±2.2 | **<0.001** |
| *Silent lacunar infarcts* | 251 (16.4%) | 63 (12.3%) | 90 (17.6%) | 98 (19.3%) | **0.003** |
| *Age, years* | 73.0±5.4 | 72.7±5.3 | 73.1±5.3 | 73.2±5.4 | 0.203 |
| *Height, cm* | 158.0±8.8 | 158.4±8.8 | 158.2±8.6 | 157.5±8.8 | 0.218 |
| *Body weight, kg* | 56.9±10.2 | 54.5±9.9 | 57.3±10.0 | 58.9±10.2 | **<0.001** |
| *Body mass index, kg/m^2^* | 22.7±3.0 | 21.6±2.5 | 22.8±2.9 | 23.7±3.3 | **<0.001** |
| *Skeletal Muscle Index, kg/ m^2^* | 6.4±1.0 | 6.4±1.0 | 6.4±1.0 | 6.5±0.9 | 0.074 |
| *Percent body fat, %* | 28.3±7.3 | 25.3±6.3 | 28.7±6.7 | 30.8±7.6 | **<0.001** |
| *Waist circumference, cm* | 86.5±9.2 | 83.5±8.1 | 86.5±8.9 | 89.6±9.6 | **<0.001** |
| *Hypertension* | 991 (64.7%) | 300 (58.7%) | 328 (64.1%) | 363 (71.5%) | **<0.001** |
| *Diabetes* | 186 (12.1%) | 46 (9.0%) | 55 (10.7%) | 85 (16.7%) | **<0.001** |
| *Hyperlipidemia* | 957 (62.5%) | 282 (55.2%) | 328 (64.1%) | 347 (68.3%) | **<0.001** |
| *Cardiovascular disease* | 67 (4.4%) | 20 (3.9%) | 18 (3.5%) | 29 (5.7%) | 0.162 |
| *Cognitive impairment* | 258 (16.9%) | 70 (13.7%) | 83 (16.2%) | 105 (20.7%) | **0.003** |
| *Smoking* |  |  |  |  |  |
| *Past smoking* | 500 (32.7%) | 150 (29.4%) | 172 (33.6%) | 178 (35.0%) | 0.053 |
| *Current smoking* | 114 (7.4%) | 36 (7.0%) | 32 (6.3%) | 46 (9.1%) | 0.223 |
| *Muscle strength, Nm* | 76.5±27.3 | 92.9±27.7 | 78.0±22.2 | 58.4±19.3 | **<0.001** |
| *Muscle strength, Nm/kg* | 133.7±37.4 | 168.2±27.5 | 134.4±20.8 | 98.4±24.0 | **<0.001** |
| *Physical activity, METs/hour/week* | 30.5 (16.5-54.2) | 33.9 (19.2-60.3) | 31.8 (16.5-47.1) | 31.8 (16.5-47.1) | **0.001** |
| *Sedentary time, hour* | 6.0±3.6 | 5.7±3.5 | 6.1±3.7 | 6.2±3.6 | **0.026** |
| *Dietary intake, kcal* | 1963.6±594.1 | 1990.1±600.2 | 1939.6±586.8 | 1961.3±595.3 | 0.335 |
| *Protein intake, g* | 83.1±30.6 | 84.9±31.6 | 82.6±30.2 | 81.8±29.9 | 0.079 |
| *Fat intake, g* | 61.6±22.0 | 63.0±22.6 | 61.1±21.6 | 60.7±21.7 | 0.131 |
| *Carbohydrate intake, g* | 242.5±82.5 | 244.2±83.5 | 240.2±81.9 | 243.1±82.2 | 0.891 |
| *Salt, g* | 12.5±4.1 | 12.5±4.0 | 12.5±4.2 | 12.5±3.9 | 0.724 |
| *Alcohol, g* | 1.0 (0.0-17.1) | 1.6 (0.0-17.6) | 1.0 (0.0-16.2) | 1.0 (0.0-16.2) | 0.229 |
| *Systolic blood pressure, mmHg* | 136.4±17.1 | 134.7±16.5 | 136.4±17.2 | 138.1±17.3 | **0.005** |
| *Diastolic blood pressure, mmHg* | 84.3±9.7 | 83.6±9.4 | 84.2±9.7 | 84.9±10.1 | 0.050 |
| *Fasting plasma insulin, μU/mL* | 4.1 (2.8-5.9) | 3.5 (2.4-5.0) | 4.1 (2.9-6.1) | 4.1 (2.9-6.1) | **<0.001** |
| *Fasting plasma glucose, mg/dL* | 96.0 (90.0-104.0) | 95.0 (90.0-102.0) | 96.0 (91.0-104.0) | 96.0 (91.0-104.0) | **<0.001** |
| *HbA1c, %* | 5.8±0.6 | 5.8±0.5 | 5.8±0.5 | 5.9±0.6 | 0.227 |
| *Total cholesterol, mg/dL* | 207.3±36.0 | 209.2±35.6 | 207.7±36.4 | 205.0±35.9 | **0.048** |
| *LDL cholesterol, mg/dL* | 122.1±30.6 | 121.6±29.1 | 122.6±30.8 | 122.1±31.9 | 0.842 |
| *HDL cholesterol, mg/dL* | 64.7±16.6 | 67.5±17.2 | 64.1±16.4 | 62.4±15.7 | **<0.001** |
| *Triglycerides, mg/dL* | 85.0 (64.0-117.0) | 78.0 (61.0-110.0) | 87.0 (65.0-122.0) | 87.0 (65.0-122.0) | **<0.001** |
| *Aspartate aminotransferase, IU/L* | 22.0 (19.0-25.0) | 23.0 (19.0-26.0) | 21.0 (19.0-25.0) | 21.0 (19.0-25.0) | 0.114 |
| *Alanine aminotransferase, IU/L* | 17.0 (13.0-21.0) | 17.0 (13.0-21.0) | 16.0 (13.0-21.0) | 16.0 (13.0-21.0) | 0.216 |
| *γ-glutamyl transferase, IU/L* | 21.0 (16.0-33.0) | 20.0 (15.0-30.0) | 21.0 (16.0-33.0) | 21.0 (16.0-33.0) | **<0.001** |
| *Serum albumin, g/dL* | 43.0±3.7 | 43.1±3.6 | 42.8±3.6 | 43.1±4.0 | 0.813 |
| *Creatinine, mg/dL* | 0.73 (0.62-0.86) | 0.7 (0.6-0.9) | 0.7 (0.6-0.9) | 0.7 (0.6-0.9) | **0.033** |
| *Total adiponectin, μg/ml* | 10.9 (7.7-15.5) | 10.9 (7.7-15.5) | 10.8 (7.6-15.2) | 10.8 (7.6-15.2) | 0.948 |
| *25-hydroxyvitamin D, ng/mL* | 19.1±5.1 | 19.4±5.2 | 19.2±5.3 | 18.8±5.0 | 0.121 |

Data are mean±SD, mean (IQR), or number (%)

*p for trend was analyzed by the Jonckheere Terpstra test (continuous variables) and Cochran-Armitage trend test (categorical variables).
