# Supplementary material for: Insulin resistance and muscle weakness are synergistic risk factors for silent lacunar infarcts: the Bunkyo Health Study
Source: Sci Rep. 2021 Oct 26;11:21093. doi: 10.1038/s41598-021-00377-5 (PMC8548532; doi:10.1038/s41598-021-00377-5)
Supplement: Supplementary file 4 — Supplementary Table S2. [file 41598_2021_377_MOESM4_ESM.docx]

**Supplementary Table 2. Associations between insulin sensitivity and silent lacunar infarcts in male subjects**

|  |  | Odds (95%CI) |  |
| --- | --- | --- | --- |
|  | Model 1 | Model 2 | Model 3 |
| Insulin sensitivity |  |  |  |
| High (≥1SD) | 1.00 | 1.00 | 1.00 |
| Medium | 2.25 (1.04-4.86) | 2.16 (0.99-4.69) | 2.10 (0.95-4.65) |
| Low (≤-1SD) | 2.83 (1.18-6.78) | 2.56 (1.06-6.18) | 2.38 (0.89-6.34) |
| ***p for trend*** | ***0.024*** | ***0.047*** | ***0.103*** |
| Muscle strength |  |  |  |
| High | 1.00 | 1.00 | 1.00 |
| Medium | 2.11 (1.21-3.67) | 2.00 (1.14-3.51) | 2.02 (1.15-3.55) |
| Low | 2.07 (1.19-3.61) | 1.96 (1.11-3.46) | 1.97 (1.11-3.49) |
| ***p for trend*** | ***0.013*** | ***0.025*** | ***0.026*** |
|  |  |  |  |
| Age (per 1 year) | 1.08 (1.04-1.13) | 1.06 (1.02-1.11) | 1.06 (1.02-1.11) |
| Smoking |  |  |  |
| Never |  | 1.00 | 1.00 |
| Past |  | 1.02 (0.63-1.67) | 1.03 (0.63-1.67) |
| Current |  | 0.61 (0.26-1.42) | 0.60 (0.26-1.42) |
| Physical activity  (per METs/hour/week) |  | 1.00 (0.99-1.00) | 1.00 (0.99-1.00) |
| Hypertension (yes) |  |  | 1.30 (0.77-2.21) |
| Diabetes (yes) |  |  | 1.06 (0.57-1.95) |
| Hyperlipidemia (yes) |  |  | 0.83 (0.53-1.31) |
| Cardiovascular disease (yes) |  |  | 1.37 (0.64-2.91) |

Model 1 was adjusted for age and sex.

Model 2 was adjusted for muscle strength or insulin sensitivity, smoking, physical activity, and incorporated model 1.

Model 3 was adjusted for hypertension, diabetes, dyslipidemia, cardiovascular disease, and incorporated model 2.
