# Supplementary material for: Insulin resistance and muscle weakness are synergistic risk factors for silent lacunar infarcts: the Bunkyo Health Study
Source: Sci Rep. 2021 Oct 26;11:21093. doi: 10.1038/s41598-021-00377-5 (PMC8548532; doi:10.1038/s41598-021-00377-5)
Supplement: Supplementary file 5 — Supplementary Table S3. [file 41598_2021_377_MOESM5_ESM.docx]

**Supplementary Table 3. Associations between insulin sensitivity and silent lacunar infarcts in female subjects**

|  |  | Odds (95%CI) |  |
| --- | --- | --- | --- |
|  | Model 1 | Model 2 | Model 3 |
| Insulin sensitivity |  |  |  |
| High (≥1SD) | 1.00 | 1.00 | 1.00 |
| Medium | 1.37 (0.76-2.48) | 1.43 (0.77-2.63) | 1.33 (0.71-2.49) |
| Low (≤-1SD) | 1.88 (0.94-3.75) | 1.88 (0.90-3.91) | 1.82 (0.83-4.02) |
| ***p for trend*** | ***0.064*** | ***0.089*** | ***0.127*** |
| Muscle strength |  |  |  |
| High | 1.00 | 1.00 | 1.00 |
| Medium | 1.13 (0.70-1.81) | 1.05 (0.65-1.71) | 1.09 (0.66-1.78) |
| Low | 1.39 (0.88-2.20) | 1.25 (0.77-2.02) | 1.20 (0.74-1.96) |
| ***p for trend*** | ***0.152*** | ***0.353*** | ***0.459*** |
|  |  |  |  |
| Age (per 1 year) | 1.11 (1.09–1.14) | 1.13 (1.09-1.17) | 1.12 (1.07-1.16) |
| Smoking |  |  |  |
| Never |  | 1.00 | 1.00 |
| Past |  | 1.07 (0.62-1.86) | 1.06 (0.61-1.86) |
| Current |  | 0.79 (0.22-2.76) | 0.92 (0.26-3.25) |
| Physical activity  (per METs/hour/week) |  | 1.00 (1.00-1.01) | 1.00 (1.00-1.01) |
| Hypertension (yes) |  |  | 2.26 (1.42-3.58) |
| Diabetes (yes) |  |  | 0.73 (0.37-1.44) |
| Hyperlipidemia (yes) |  |  | 0.89 (0.58-1.35) |
| Cardiovascular disease (yes) |  |  | 2.84 (1.16-6.96) |

Model 1 was adjusted for age and sex.

Model 2 was adjusted for muscle strength or insulin sensitivity, smoking, physical activity, and incorporated model 1.

Model 3 was adjusted for hypertension, diabetes, dyslipidemia, cardiovascular disease, and incorporated model 2.
